# Draft genome sequence of *Bacillus pumilus* SP1, a soil isolate with antagonistic activity toward *Staphylococcus epidermidis*

**DOI:** 10.1128/mra.01424-25

**Published:** 2026-03-10

**Authors:** Sean Pauly, Fatemah Hermes

**Affiliations:** 1 College of Business Administration, University of Missouri - St. Louis 14716, St. Louis, Missouri, USA; 2 Department of Basic Sciences, University of Health Sciences and Pharmacy 576777, St. Louis, Missouri, USA; Fluxus Inc., Sunnyvale, California, USA

**Keywords:** soil microbiology, antagonism, *Bacillus*

## Abstract

A *Bacillus pumilus* soil isolate displayed antagonistic activity against *Staphylococcus epidermidis* ATCC 14990. Here, we present a draft genome sequence for this isolate.

## ANNOUNCEMENT

Soil microbes drive carbon, nitrogen, and phosphorus cycling and modulate mineral availability (1). They support plant growth through chemical signaling and protect plants against pests and pathogens through antibiotic production (2). The central role of these microbes in soil health directly links them to the well-being of animals and humans (3, 4). Maintaining healthy soils reduces the need for fertilizers and therefore contributes to climate change mitigation (5). Many of these microorganisms have valuable industrial applications, further underscoring their importance (6).

**Fig 1:**
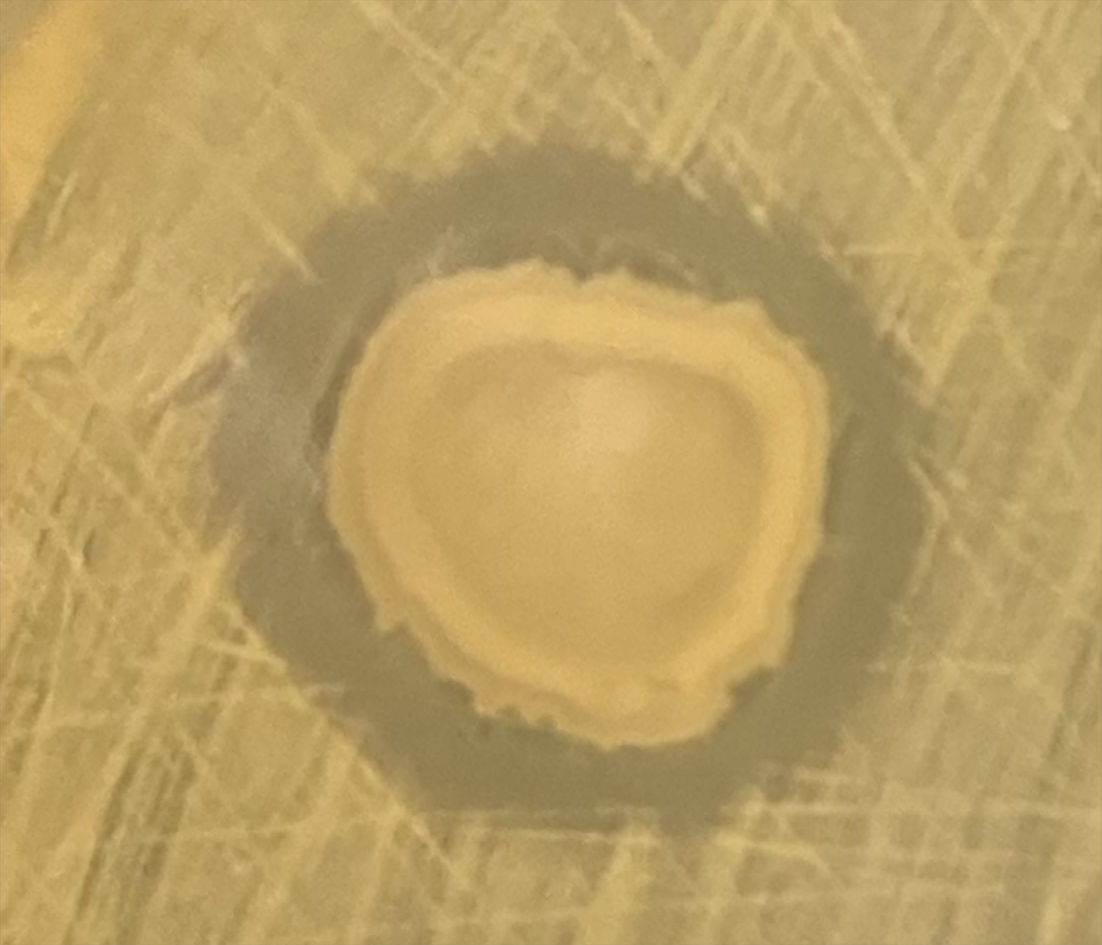
SP1 antagonism of *S. epidermidis* ATCC 14990. Both strains were grown overnight in LB, Miller broth with aeration; 50 µL of the *S. epidermidis* culture was spread on BHI agar, and 10 µL of the SP1 culture was spotted on the lawn. The plate was incubated overnight at 33°C. This image shows SP1 growth surrounded by a clear zone, then the *S. epidermidis* lawn.

As part of a microbiology course at the University of Health Sciences and Pharmacy, a sample of surface soil was collected in a sterile 50-mL tube in spring 2025 from the St. Louis area. After serial dilution in sterile water of a suspension of 1 g of the soil, aliquots were plated on brain heart infusion (BHI) agar plates containing 25 µg/mL cycloheximide and incubated at 33°C for 48 h. Several of the colonies that appeared were colony-purified by repeated quadrant streaking and then tested for antagonistic behavior against a collection of lab strains as described in the legend to Fig. 1. Isolate SP1 inhibited the growth of *Staphylococcus epidermidis* ATCC 14990 (Fig. 1) and was thus chosen for genome sequencing. The bacterium was grown to mid-log phase in Luria-Bertani, Miller (LB) broth (4 h) at 37°C with shaking at 180 rpm. About 6 × 10^9^ cells were collected, washed with saline phosphate buffer, and resuspended in 500 µL DNA/RNA stabilization solution from Zymo. The suspension was sent to Plasmidsaurus for genomic DNA extraction, library prep, and sequencing.

DNA extraction was conducted using the Zymo Quick DNA Miniprep Plus Kit. DNA quantity was assessed using Qubit. Library prep was performed using the ultra-long DNA sequencing kit v14 from Oxford Nanopore Technologies (ONT). Sequencing was conducted on a PromethION instrument using an R10.4.1 flow cell. Base calling, adapter trimming, and read filtering were performed by ONT’s Dorado v.0.5.0 with quality filter set to Q10. A total of 133,214 reads were obtained.

The 16S rRNA gene was parsed using Mash v2.3 (7) and Sourmash v4.9.4 (8) and identified the bacterium as *Bacillus pumilus. De novo* genome assembly was carried out on the BV-BRC platform (9) using Canu v2.0 (10) with an estimated genome size set to 5 Mbp and target genome coverage set to 100×. The assembly was polished with four iterations of Racon v1.5.0 (11). Assembly quality was assessed using QUAST v5.2.0 (12). Annotation was automatically performed by the NCBI Prokaryotic Genome Annotation Pipeline (PGAP) v6.10 (13). CheckM completeness was 99.9% (14). Unless otherwise noted, all software settings were set to default.

The SP1 genome is 4067923 bp long and consists of 12 contigs with an average depth of coverage of 118.73×, N50 and N90 = 3,845,078 bp, and 41.08% GC content. Contig 7 may be part of a plasmid as it has almost 2× coverage relative to the longest read. There are a total of 4,195 predicted genes in the genome. Information about the assembly and annotation is summarized in Table 1.

**TABLE 1:** Summary of *B. pumilus* SP1 genome assembly and annotation

Contig	Length	Coverage normalized to the longest contig	No. of predicted genes
1	3,845,078	1	3,954
2	28,793	0.26	30
3	27,188	0.21	28
4	17,103	0.48	19
5	42,965	0.48	47
6	14,430	0.14	17
7	16,551	1.9	20
8	20,736	0.34	22
9	15,597	0.12	18
10	12,181	0.28	15
11	17,633	0.39	15
12	9,668	0.15	10

## Data Availability

This work is registered at the NCBI under BioProject PRJNA1372794. The BioSample identifier of SP1 is SAMN53626511. The raw sequence data file is SRX31311881. The Whole Genome Shotgun project has been deposited in GenBank under the accession no. JBSQHT000000000. The version described in this paper is the first version, JBSQHT01000000

## References

[1] Iqbal S, Begum F, Nguchu BA, Claver UP, Shaw P. 2025. The invisible architects: microbial communities and their transformative role in soil health and global climate changes. Environ Microbiome 20:36. doi:10.1186/s40793-025-00694-6 40133952 PMC11938724

[2] Wang X, Chi Y, Song S. 2024. Important soil microbiota’s effects on plants and soils: a comprehensive 30-year systematic literature review. Front Microbiol 15:1347745. doi:10.3389/fmicb.2024.1347745 38591030 PMC10999704

[3] Banerjee S, van der Heijden MGA. 2023. Soil microbiomes and one health. Nat Rev Microbiol 21:6–20. doi:10.1038/s41579-022-00779-w 35999468

[4] Sabater C, Neacsu M, Duncan SH. 2025. Harnessing beneficial soil bacteria to promote sustainable agriculture and food security: a one health perspective. Front Microbiol 16:1638553. doi:10.3389/fmicb.2025.1638553 41048500 PMC12491171

[5] Kiprotich K, Muema E, Wekesa C, Ndombi T, Muoma J, Omayio D, Ochieno D, Motsi H, Mncedi S, Tarus J. 2025. Unveiling the roles, mechanisms and prospects of soil microbial communities in sustainable agriculture. Discov Soil 2:10. doi:10.1007/s44378-025-00037-4

[6] Sharma N, Ahlawat YK, Stalin N, Mehmood S, Morya S, Malik A, H M, Nellore J, Bhanot D. 2024. Microbial enzymes in industrial biotechnology: sources, production, and significant applications of lipases. J Ind Microbiol Biotechnol 52:kuaf010. doi:10.1093/jimb/kuaf010 40328460 PMC12094072

[7] Ondov BD, Treangen TJ, Melsted P, Mallonee AB, Bergman NH, Koren S, Phillippy AM. 2016. Mash: fast genome and metagenome distance estimation using MinHash. Genome Biol 17:132. doi:10.1186/s13059-016-0997-x 27323842 PMC4915045

[8] Pierce NT, Irber L, Reiter T, Brooks P, Brown CT. 2019. Large-scale sequence comparisons with sourmash. F1000Res 8:1006. doi:10.12688/f1000research.19675.1 31508216 PMC6720031

[9] Olson RD, Assaf R, Brettin T, Conrad N, Cucinell C, Davis JJ, Dempsey DM, Dickerman A, Dietrich EM, Kenyon RW, et al.. 2023. Introducing the bacterial and viral bioinformatics resource center (BV-BRC): a resource combining PATRIC, IRD and ViPR. Nucleic Acids Res 51:D678–D689. doi:10.1093/nar/gkac1003 36350631 PMC9825582

[10] Koren S, Walenz BP, Berlin K, Miller JR, Bergman NH, Phillippy AM. 2017. Canu: scalable and accurate long-read assembly via adaptive k-mer weighting and repeat separation. Genome Res 27:722–736. doi:10.1101/gr.215087.116 28298431 PMC5411767

[11] Vaser R, Sović I, Nagarajan N, Šikić M. 2017. Fast and accurate de novo genome assembly from long uncorrected reads. Genome Res 27:737–746. doi:10.1101/gr.214270.116 28100585 PMC5411768

[12] Gurevich A, Saveliev V, Vyahhi N, Tesler G. 2013. QUAST: quality assessment tool for genome assemblies. Bioinformatics 29:1072–1075. doi:10.1093/bioinformatics/btt086 23422339 PMC3624806

[13] Tatusova T, DiCuccio M, Badretdin A, Chetvernin V, Nawrocki EP, Zaslavsky L, Lomsadze A, Pruitt KD, Borodovsky M, Ostell J. 2016. NCBI prokaryotic genome annotation pipeline. Nucleic Acids Res 44:6614–6624. doi:10.1093/nar/gkw569 27342282 PMC5001611

[14] Parks DH, Imelfort M, Skennerton CT, Hugenholtz P, Tyson GW. 2015. CheckM: assessing the quality of microbial genomes recovered from isolates, single cells, and metagenomes. Genome Res 25:1043–1055. doi:10.1101/gr.186072.114 25977477 PMC4484387

